# Dual-Pigmented Rice: A Dual-Pigmented Whole Grain with Enhanced Nutritional and Antioxidant Properties

**DOI:** 10.3390/foods15193530

**Published:** 2026-10-02

**Authors:** Zijin Weng, Wenjun Wei, Faming Dong, Yilong Yao, Yanhua Li, Qinglu Zhang, Xu Li, Yuqiong Zhou, Yongjun Lin, Hao Chen

**Affiliations:** National Key Laboratory of Crop Genetic Improvement, Hubei Hongshan Laboratory, Huazhong Agricultural University, Wuhan 430070, China; chris911@webmail.hzau.edu.cn (Z.W.);

**Keywords:** *Oryza sativa*, dual-pigmented rice, functional food, proanthocyanidins, anthocyanins

## Abstract

Pigmented rice is valued for its abundant natural bioactive compounds and potential health-promoting properties. In this study, the black rice cultivar Huamoxiang No. 5 (HMX) was used to develop three genetically similar rice lines with distinct pericarp pigmentation. Among them, BRHMX (a dual-pigmented rice accumulating both anthocyanins and proanthocyanidins) and RHMX (a red rice accumulating proanthocyanidins) were generated through hybridization, successive backcrossing, and marker-assisted selection, whereas the white rice line WHMX was derived from a spaceflight-induced mutant of HMX. Comparative analyses revealed that differences in pericarp pigmentation had negligible effects on grain morphology and major nutritional components, including starch, protein, and amino acids. Compared with HMX, BRHMX maintained more than 80% of the anthocyanin content of HMX, while its proanthocyanidin content was 103% higher than that of RHMX. Furthermore, BRHMX exhibited the highest total phenolic content and the highest levels of 31 soluble phenolic acids, together with the strongest chemical antioxidant capacity, as determined by ABTS and FRAP assays. Untargeted metabolomic profiling showed that flavonoids and soluble phenolic acids were the major metabolite classes associated with pericarp color divergence. Integrated metabolomic and transcriptomic analyses further suggested a potential reallocation of metabolic flux between anthocyanin and proanthocyanidin biosynthetic pathways during early pericarp development. Cooking generally reduced the contents of anthocyanins, proanthocyanidins, and total phenolics, as well as chemical antioxidant capacity, although the magnitude of these changes varied among the four lines. Collectively, these findings demonstrate that the newly developed dual-pigmented rice line combines characteristics associated with conventional black and red rice, providing a valuable genetic resource for whole-grain rice product development.

## 1. Introduction

Rice is a staple food for more than half of the world’s population, especially in Asia where it is consumed as a daily dietary component [1,2]. Breeding preferences for rice, particularly in regions where higher incomes and food security have alleviated survival-driven priorities, have shifted from a primary focus on high yield and resistance to biotic and abiotic stresses to increasing attention to quality and nutritional value [1]. Among rice varieties, pigmented rice, with black and red rice as the two major categories, has attracted considerable attention for its unique nutritional properties and health benefits, particularly its richness in bioactive flavonoids and phenolic acid compounds and its potential health-promoting effects associated with its strong antioxidant activity [3,4,5]. Black rice primarily accumulates anthocyanins in the pericarp, whereas red rice is characterized by the accumulation of proanthocyanidins. The predominant anthocyanins in rice are cyanidin-3-*O*-glucoside (C3G) and peonidin-3-*O*-glucoside (P3G), while proanthocyanidins mainly consist of polymers derived from catechin and epicatechin units, with proanthocyanidin B2 being the most abundant form [6]. Both anthocyanins and proanthocyanidins are important plant-derived antioxidants with reported biological activities, including free radical scavenging, anti-inflammatory effects, cardiovascular protection, and regulation of metabolic disorders [7,8,9]. Therefore, these compounds have attracted increasing attention as promising components for functional food development and nutritional improvement of cereal crops.

In plants, the biosynthesis of anthocyanin and proanthocyanidin shares many of the same upstream biosynthetic pathways, both originating from the phenylpropanoid pathway [10]. Phenylalanine is first deaminated by phenylalanine ammonia-lyase (PAL) to form p-coumaric acid, which is subsequently converted into p-coumaroyl-CoA. Together with malonyl-CoA, p-coumaroyl-CoA is then condensed by chalcone synthase (CHS) to produce tetrahydroxychalcone, which is isomerized by chalcone isomerase (CHI) to form naringenin. Naringenin is hydroxylated by flavanone 3-hydroxylase (F3H) to form dihydrokaempferol, which can be further modified by flavonoid 3′-hydroxylase (F3′H) and flavonoid 3′,5′-hydroxylase (F3′5′H) to yield dihydroquercetin and dihydromyricetin, respectively. These intermediates are reduced by dihydroflavonol 4-reductase (DFR) to produce leucoanthocyanidins. At this key branching point, leucoanthocyanidins have two major fates: they can be converted into anthocyanidins by anthocyanidin synthase (ANS), which are subsequently glycosylated by UDP-glucose flavonoid 3-*O*-glucosyltransferase (UFGT) to form stable anthocyanins; alternatively, leucoanthocyanidins can be reduced by leucoanthocyanidin reductase (LAR) to generate catechin [11,12]. In addition, anthocyanidins can be reduced by anthocyanidin reductase (ANR) to produce epicatechin. Finally, catechin and epicatechin undergo polymerization to generate proanthocyanidins [13,14].

The expression of anthocyanin and proanthocyanidin biosynthetic genes, as well as pigment accumulation in specific plant tissues, is mainly regulated by the MYB-bHLH-WD40 (MBW) transcriptional complex [15]. Wild rice typically exhibits red pericarp pigmentation due to proanthocyanidin biosynthesis. A loss-of-function mutation in the bHLH gene *Rc* prevents proanthocyanidin biosynthesis, leading to the conversion of red rice into white rice [16,17]. During rice domestication, the white pericarp trait was preferentially selected, leading to the predominance of white-pericarp cultivars among modern rice varieties. In contrast, black rice originated from a gain-of-function mutation in another bHLH gene, *Kala4*, in which an approximately 11 kb upstream insertion activated a previously non-expressed gene and enabled anthocyanin biosynthesis in the pericarp [12,18]. A survey of 533 rice accessions conducted in China showed that 85.4% were white rice, 13.5% were red rice, and only 1.1% (six accessions) were black rice [12,19]. Among these black rice accessions, only one carried a functional *Rc* allele, allowing the simultaneous accumulation of anthocyanins and proanthocyanidins in the pericarp, thereby representing the rare dual-pigmented rice phenotype [19,20]. This finding indicates that dual-pigmented rice appears to represent an extremely rare pigmentation type, occurring at a substantially lower frequency than white, red, or black rice [12,19,20].

Previous studies have compared the nutritional profiles and health benefits of rice with different pericarp colors [6,21,22]. Proanthocyanidins and other flavonoids have been shown to exhibit stronger radical-scavenging activity than vitamin C and vitamin E [23]. Red rice generally exhibits higher antioxidant capacity than white rice, with reported values up to 3-fold higher, which is mainly attributed to the accumulation of proanthocyanidins and related phenolic compounds [24]. Black rice typically contains higher levels of total phenolics and flavonoids than red and white rice, contributing to its enhanced antioxidant properties [25,26,27]. Moreover, rice varieties capable of accumulating both anthocyanins and proanthocyanidins have been reported to exhibit stronger antioxidant activity than conventional black rice, suggesting the potential nutritional advantages of dual-pigmented rice [24]. Therefore, developing and utilizing dual-pigmented rice resources may provide new opportunities for improving the diversity and functional value of whole-grain rice products.

However, previous comparisons have largely been conducted among rice varieties with distinct genetic backgrounds, making it difficult to distinguish the effects of pericarp pigmentation from confounding genetic variation. In this study, four genetically similar Huamoxiang lines derived from the high-quality whole-grain black rice cultivar Huamoxiang No. 5 (HMX) were used as a comparative system to evaluate pigmentation-associated differences. Grain morphology and major nutritional components, including starch, protein, and amino acids, as well as lipid composition, were systematically assessed among these lines. High-performance liquid chromatography (HPLC) and liquid chromatography–tandem mass spectrometry (LC–MS/MS) were used to quantify anthocyanins, proanthocyanidins, and 50 soluble phenolic acids. These analyses were further complemented by untargeted metabolomic profiling and transcriptome analysis of pericarps collected at 10 days after pollination (10 DAP) to characterize metabolic and transcriptional changes associated with different pericarp pigmentation patterns. In addition, the contents of anthocyanins, proanthocyanidins, and total phenolics, together with chemical antioxidant capacity, were evaluated in cooked rice to assess changes in these bioactive components and antioxidant properties following cooking. This study provides a comprehensive evaluation of the nutritional composition, bioactive metabolites, and chemical antioxidant capacity of dual-pigmented rice, both before and after cooking, and establishes a scientific basis for its utilization in whole-grain food development.

## 2. Materials and Methods

### 2.1. Rice Materials

HMX is a black rice variety developed by our research group. It is characterized by excellent eating quality and is rich in anthocyanins [28]. However, HMX does not contain proanthocyanidins because it carries a non-functional *Rc* allele.

HMX was used as the female parent and the red rice line HHZ-Rc, an *Rc* single-gene introgression line in the Huanghuazhan (HHZ) background, was used as the male parent for hybridization. Following successive backcrossing combined with marker-assisted selection and subsequent self-pollination, two lines with the highest genetic similarity to HMX were selected from the segregating progeny, including the dual-pigmented line BRHMX and the red-pigmented line RHMX. BRHMX carries functional alleles of both *Kala4* and *Rc*, whereas RHMX contains a functional *Rc* allele but a non-functional *Kala4* allele. In addition, the white rice line WHMX was obtained from a spaceflight-induced mutant of HMX and subsequently stabilized through six generations of self-pollination. WHMX carries non-functional alleles of both *Kala4* and *Rc*. The detailed breeding scheme is presented in Figure S1.

### 2.2. Field Experimental Design and Crop Management

The four Huamoxiang lines with distinct pericarp colors were cultivated in early May 2024 at the Rice Experimental Station of Huazhong Agricultural University, Wuhan, China (114°21′28″ E, 30°28′1″ N). A randomized complete block design was applied, with three biological replicates for each line. The planting order of the four lines was determined by random drawing, resulting in a total of 12 plots arranged in a 3 × 4 layout. Each plot consisted of 16 plants arranged in a 4 × 4 pattern, with 20 cm row spacing and 16 cm plant spacing. Each plot was considered an experimental unit and represented one independent biological replicate. At harvest, the 16 plants within each plot were harvested and pooled to generate one biological sample for subsequent analyses. Two rows of purple-leafed white rice were planted around each plot as border rows to minimize edge effects.

All four lines were grown under the same field conditions and received identical and uniform agronomic management throughout the growing season. Fertilization was applied uniformly across all plots. Before transplanting, NPK 17-17-17 fertilizer was applied at 375 kg/ha, followed by urea at 150 kg/ha 7 d after transplanting. No additional fertilizer was applied to individual plots or areas with poor growth. Irrigation was managed according to rice growth stage, with moist or shallow-water conditions maintained during vegetative growth, periodic drainage during tillering, and alternating wetting and drying from heading to grain filling. Pest management was conducted uniformly across all plots. Rice leaf folder and stem borers were each controlled once using 10% triflumezopyrim at 180 mL/ha in combination with 16% emamectin benzoate–indoxacarb at 180 mL/ha. Brown planthoppers were controlled once using 10% triflumezopyrim at 180 mL/ha. No routine fungicides were applied, and no severe insect or fungal disease outbreaks that could substantially affect plant growth or grain development were observed during the experimental period. Plants were harvested in late September 2024.

### 2.3. Genotyping of Breeding Materials

During the breeding and selection process, genomic DNA was extracted separately from each candidate individual plant at each generation using an alkaline lysis method. No pooled samples were used for genotyping. Individual plants were first genotyped for *Rc* using Kompetitive Allele-Specific PCR (KASP) assays, and plants carrying the desired genotype were subsequently subjected to genome-wide SNP genotyping to evaluate their genetic similarity to the recurrent parent HMX. At each generation, one individual plant with the desired genotype and the highest genetic similarity to HMX was selected for further propagation.

Genotyping of BRHMX and RHMX was performed according to Lu et al. [29] with minor modifications. Genomic DNA was extracted from young rice leaves using an alkaline lysis method. In brief, fresh leaf samples in 96-well plates were ground with NaOH, neutralized with Tris-HCl, and diluted 10-fold for direct use as PCR templates.

KASP assays for *Rc* were performed using primers designed with SNPWay (www.snpway.com). Each 5 μL reaction contained 2.5 μL of 2× KASP Master Mix, 0.3 μL of primer mix, and approximately 1.5 μL of template DNA (~50 ng/μL). The PCR program consisted of an initial denaturation at 94 °C for 1 min, followed by 10 touchdown cycles (94 °C for 20 s and 66–58 °C for 60 s, decreasing by 0.8 °C per cycle), 35 amplification cycles (94 °C for 20 s and 57 °C for 60 s), and a final hold at 25 °C for 1 min. Fluorescence signals were detected using a BMG Omega F instrument (BMG Labtech, Offenburg, Germany), and genotype calling was performed with LGC KlusterCaller™ software (version 2.7.1). Primers designed in the present study are listed in Table S1.

Genome-wide SNP genotyping among the four lines was performed by GreenFaFa Co., Ltd. (Wuhan, China) using the SLYm1R high-density rice SNP array. The array was developed based on Illumina microarray technology using the *Nipponbare* reference genome (MSU v7.0) and contains 31,753 probe loci, including 31,704 loci with assigned genomic positions and 49 other probes. Genetic similarity among the four lines was calculated as the proportion of probe loci showing identical genotypes relative to the total number of detected probe loci.

### 2.4. Sample Preparation

Each plot was harvested independently, and grains collected from the 16 plants within each plot were pooled and considered as one biological replicate. After harvesting and threshing, rice samples were freeze-dried and stored at −20 °C until further analysis. Before analysis, samples were equilibrated to room temperature and dehulled using a rice huller (Sinograin, Shanghai, China). Grain appearance traits were evaluated using an SC-E rice appearance quality analyzer (Wseen, Hangzhou, China). Polished rice samples were prepared by removing the bran and aleurone layers using a rice polisher (Kett, Tokyo, Japan). Both whole-grain rice and polished rice samples were subsequently ground into powder using a cyclone mill (FOSS, Hillerød, Denmark). Unless otherwise indicated, all measurements were performed using three biological replicates.

For the cooking treatment, 30 g of whole-grain rice samples was weighed into aluminum containers, and distilled water was added at a rice:water ratio of 1:1.5 (*w*/*v*). The containers were covered and placed in an electric steamer (Joyoung, Jinan, China), operated at 1200 W, for 40 min. After steaming, the cooked rice samples were allowed to cool naturally to room temperature and then freeze-dried for 2 d. The freeze-dried samples were ground into powder using the same procedure described above for the uncooked whole-grain rice samples and stored at −20 °C until further analysis. Three independent biological replicates were prepared for each Huamoxiang line.

### 2.5. Extraction and Quantification of Anthocyanins

The total anthocyanin content in each sample was calculated as the sum of C3G and P3G concentrations [3]. Briefly, 0.05 g of whole-grain rice powder was extracted with 1 mL of methanol:water:hydrochloric acid (80:20:1, *v*/*v*/*v*) in a 2.0 mL centrifuge tube. The samples were extracted at room temperature on a horizontal shaker at 170 rpm for 24 h and then centrifuged at 12,000 rpm for 10 min at room temperature. The resulting supernatants were filtered through a 0.22 μm membrane filter before HPLC analysis. Anthocyanin contents were determined using an Agilent 1260 HPLC system (Agilent, Santa Clara, CA, USA) equipped with an Agilent ZORBAX SB-Aq C18 column (4.6 mm × 250 mm, 5 μm). The mobile phases consisted of 0.1% formic acid in water (solvent A) and methanol (solvent B), with detection performed at 520 nm for the simultaneous determination of C3G and P3G [3]. Quantification was performed using C3G and P3G as external standards.

### 2.6. Extraction and Quantification of Proanthocyanidins

Proanthocyanidins were extracted according to the method described by Abeynayake et al. [30] with minor modifications. Briefly, 0.05 g of whole-grain rice powder was extracted with acetone:water:ascorbic acid (70:30:0.1, *v*/*v*/*w*) in an ice-water bath with ultrasonic treatment for 30 min at 500 W. After centrifugation at 12,000 rpm for 10 min, the supernatant was collected, and the extraction procedure was repeated once under the same conditions. The combined extracts were evaporated to dryness under a nitrogen stream (TUOHE, Shanghai, China) and redissolved in 1 mL of 80% methanol to obtain the proanthocyanidin extract. Proanthocyanidin content was determined using the DMACA (4-dimethylaminocinnamaldehyde) colorimetric method. Briefly, 50 μL of the extract was mixed with 150 μL of DMACA reagent (0.1%, *w*/*v*, DMACA in methanolic HCl) in a microplate, and the reaction was monitored using a Spark multimode microplate reader (Tecan, Männedorf, Switzerland). Absorbance at 640 nm was recorded continuously for 30 min, and the maximum absorbance value was used for quantification. For samples containing anthocyanins, control reactions containing methanolic HCl without DMACA were performed to correct for potential interference from anthocyanins [31]. Proanthocyanidin B2 was used as the external standard for quantification.

### 2.7. Determination of Total Phenolics and Soluble Phenolic Acids

Total phenolic content was determined according to a modified method described by Tang et al. [32]. The anthocyanin and proanthocyanidin extracts were mixed at a 1:1 (*v*/*v*) ratio to improve the extraction efficiency of phenolic compounds with different polarities. The mixed extracts were reacted with a commercial total phenolic assay kit (Sangon, Shanghai, China), and absorbance was measured at 760 nm using a multimode microplate reader. Gallic acid was used as the external standard for quantification.

Soluble phenolic acids were determined according to a modified protocol described by Shao et al. [33]. Briefly, 0.20 g of whole-grain rice powder was extracted twice with 80% methanol. The combined extracts were evaporated to dryness under a nitrogen stream and reconstituted in 100 μL of methanol (pH 2.0). The remaining residue was further subjected to acid–base extraction. After evaporation under nitrogen, the extract was reconstituted in 100 μL of methanol (pH 2.0). The two fractions were combined and analyzed using an LC-MS/MS Triple QTRAP 5500+ system (AB SCIEX, Framingham, MA, USA) according to the method described by Chen et al. [34].

### 2.8. Determination of Total Antioxidant Capacity

Total antioxidant capacity was evaluated using ABTS and FRAP assays. For both assays, the sample solution was prepared by mixing the proanthocyanidin and anthocyanin extracts at a 1:1 (*v*/*v*) ratio.

The ABTS radical-scavenging capacity was determined using a commercial ABTS assay kit (Sangon, China), based on the ABTS radical cation decolorization assay described by Re et al. [35]. Briefly, 10 μL of the sample solution was mixed with 190 μL of ABTS working solution and incubated at room temperature in the dark for 20 min. Absorbance was measured at 760 nm using a multimode microplate reader. Trolox was used as the external standard for quantification.

FRAP antioxidant capacity was measured using a FRAP assay kit (Solarbio, Beijing, China) [36]. Briefly, 30 μL of the sample solution was mixed with 900 μL of FRAP reagent and incubated at room temperature for 10 min. Absorbance was measured at 593 nm, and FeSO_4_ was used as the external standard for quantification.

### 2.9. Amylose Content

Amylose content in polished rice was determined according to the method described by Tan et al. [37]. Briefly, starch was extracted using NaOH solution and subsequently reacted with iodine reagent. The absorbance of the resulting complex was measured at 620 nm using a multimode microplate reader (Tecan, Switzerland). Amylose content was calculated based on a standard curve (y = 0.0117x + 0.0812, R^2^ = 0.999).

### 2.10. Total Starch Content

Total starch content in polished rice was determined using the K-TSTA Total Starch (AA/AMG) Assay Kit (Megazyme, Bray, Ireland) according to the manufacturer’s instructions [38]. Briefly, 0.10 g of rice flour was dispersed in 80% ethanol, treated with 2 M KOH, and enzymatically hydrolyzed with α-amylase and amyloglucosidase at 50 °C for 30 min. After adjusting the volume, the supernatant reacted with GOPOD reagent. Absorbance was measured at 510 nm, and starch content was calculated based on glucose equivalents.

### 2.11. Total Protein and Amino Acid Content

Total protein content in whole-grain rice was determined using the Kjeldahl method, as commonly applied for rice protein analysis [39], with an automated Kjeldahl analyzer (FOSS, Denmark). Briefly, 0.50 g of rice flour was digested at 420 °C with a catalyst mixture of potassium sulfate and copper sulfate (9:1, *w*/*w*). Nitrogen recovery was validated using ammonium sulfate, with a recovery rate of 99.5–100.5%. Protein content was calculated based on the measured nitrogen content.

Amino acid composition was determined according to the method described by Huang et al. [40] with a minor modification. Briefly, 0.10 g of whole-grain rice powder was hydrolyzed with 6 M HCl, concentrated under vacuum, and analyzed using an LA8080 automatic amino acid analyzer (Hitachi, Tokyo, Japan).

### 2.12. Fatty Acid Content

Fatty acid composition in whole-grain rice was determined by gas chromatographic analysis of fatty acid methyl esters (FAMEs). Briefly, 0.20 g of rice flour was mixed with 4.5 mL of sulfuric acid–methanol solution (5:95, *v*/*v*) containing 0.43 mg of margaric acid (C17:0, dissolved in chloroform) as an internal standard. The mixture was incubated at 85 °C for 2.5 h and then cooled to room temperature. FAMEs were extracted with 2 mL of hexane. After centrifugation, 1 mL of the upper phase was transferred into an amber vial for gas chromatographic analysis. Individual fatty acids were identified based on their retention times (RTs), which were periodically calibrated using FAME standards. Margaric acid (C17:0) was used as an internal standard, and fatty acid concentrations were semiquantitatively estimated as C17:0 equivalents, assuming a uniform response factor of 1.0 for all fatty acids, as is common in routine GC-FID analysis where response factors are often close to unity. Fatty acid profiles were determined using a GC-2030 gas chromatograph (Shimadzu, Kyoto, Japan) according to the method described by Zhou et al. [41].

### 2.13. Transcriptome Analysis

Transcriptome sequencing was performed on 10 DAP pericarps from the four Huamoxiang lines with distinct pericarp colors by SMART GENOMICS Technology Co., Ltd. (Tianjin, China). Three biological replicates were included for each line. Libraries were sequenced on the DNBSEQ-T7 platform (BGI, Shenzhen, China) using the PE150. Raw data analysis was performed on the server of the National Key Laboratory of Crop Genetic Improvement, Huazhong Agricultural University. Fastp v0.23.4 was used for quality control and filtering [42]. Reads were aligned to the *Oryza sativa Nipponbare* IRGSP-1.0 reference genome using HISAT2 v2.2.1 [43]. Gene-level read counts were obtained using FeatureCounts from the Subread v2.0.2 package [44]. Differential expression analysis was performed using DESeq2 v1.46.0 in R [45]. Genes with adjusted *p*-values (FDR) ≤ 0.05 were considered differentially expressed. Kyoto Encyclopedia of Genes and Genomes (KEGG) pathway enrichment analysis was conducted using clusterProfiler v4.14.4 [46,47,48]. Transcription factor annotations were obtained from PlantTFDB (https://planttfdb.gao-lab.org/). Pearson’s correlation coefficients were calculated using R’s built-in cor function. Data visualization was performed in R v4.4.1 using the pheatmap and corrplot packages. Network topology was visualized using Cytoscape v3.10.3.

### 2.14. Untargeted Metabolomics Analysis

Untargeted metabolomic profiling of 10 DAP pericarps and mature seeds of the four lines was conducted by Biotree Biomedical Technology Co., Ltd. (Shanghai, China), with three biological replicates per sample. Chromatographic analysis was performed using a Vanquish UPLC system (Thermo Fisher Scientific, Waltham, MA, USA), coupled with an Orbitrap Exploris 120 mass spectrometer (Thermo Fisher Scientific, USA). A Phenomenex Kinetex C18 column (2.1 mm × 50 mm, 2.6 μm) was used. Standard protocols were followed.

Raw metabolomic data processing and metabolite annotation were performed by the service provider. Data preprocessing included outlier filtering, missing-value filtering and imputation, and normalization using internal standards. The metabolite annotation workflow comprised four confidence levels. Level 1 metabolites were annotated using the service provider’s in-house reference-standard database established with authentic standards, based on matching RT, MS1, and MS2 information, with matching criteria of 15 ppm for MS1, 25 ppm for MS2, and 30 s for RT. Level 2 metabolites were putatively annotated based on MS1 and MS2 spectral matching against public databases. Only metabolites annotated at Level 1 and Level 2 were included in subsequent analyses. The numbers of metabolites in each category were summarized and visualized using GraphPad Prism v10.4.0. Principal component analysis (PCA), hierarchical clustering analysis (HCA), differential metabolite screening, and KEGG enrichment were conducted using MetaboAnalystR v4.0 and MetaboAnalyst 6.0 [49,50]. Orthogonal partial least squares-discriminant analysis (OPLS-DA) was conducted using SIMCA v18.0.1. Model quality was evaluated using seven-fold cross-validation, with R^2^Y and Q^2^ used to assess model fit and predictive ability, respectively. To evaluate potential model overfitting, permutation testing was performed with 200 permutations. Volcano plots, Venn diagrams, and KEGG enrichment bubble charts were generated using R v4.4.1.

### 2.15. Statistical Analysis

Analysis of variance (ANOVA) and Tukey’s HSD tests were performed using GraphPad Prism v10.4.0 (GraphPad, San Diego, CA, USA). Different letters (a, b, c, d) indicate statistically significant differences at *p* < 0.05.

## 3. Results

### 3.1. Development of Genetically Similar Huamoxiang Lines with Different Pericarp Colors

To develop genetically similar Huamoxiang rice lines with different pericarp colors, black rice HMX was used as the female parent, and red rice HHZ-Rc, an *Rc* single-gene introgression line in the Huanghuazhan background, served as the male parent for hybridization. The progeny was backcrossed with HMX for three consecutive generations to generate the BC_3_F_1_ population. During the hybridization and backcrossing processes, PCR screening was performed to identify recombinant individuals carrying recombination events on both sides of the *Rc* locus within approximately 100 kb of the upstream and downstream flanking regions. Subsequently, *Rc* genotypes were determined using KASP-based SNP markers, and *Kala4* allelic variation was identified by conventional PCR in the selfed progeny. Genome-wide SNP chip analysis was further conducted to evaluate genetic similarity across the genome and select individuals with the highest genetic similarity to HMX. The selected BC_3_F_1_ individuals were self-pollinated for two generations to develop the dual-pigmented line BRHMX. Meanwhile, selected BC_3_F_1_ plants were further backcrossed once with HMX, followed by one generation of self-pollination, to generate the red-pigmented line RHMX. WHMX was derived from a spaceflight-induced mutant of HMX and was genetically stabilized after six generations of self-pollination (Figure S1).

**Figure 1:**
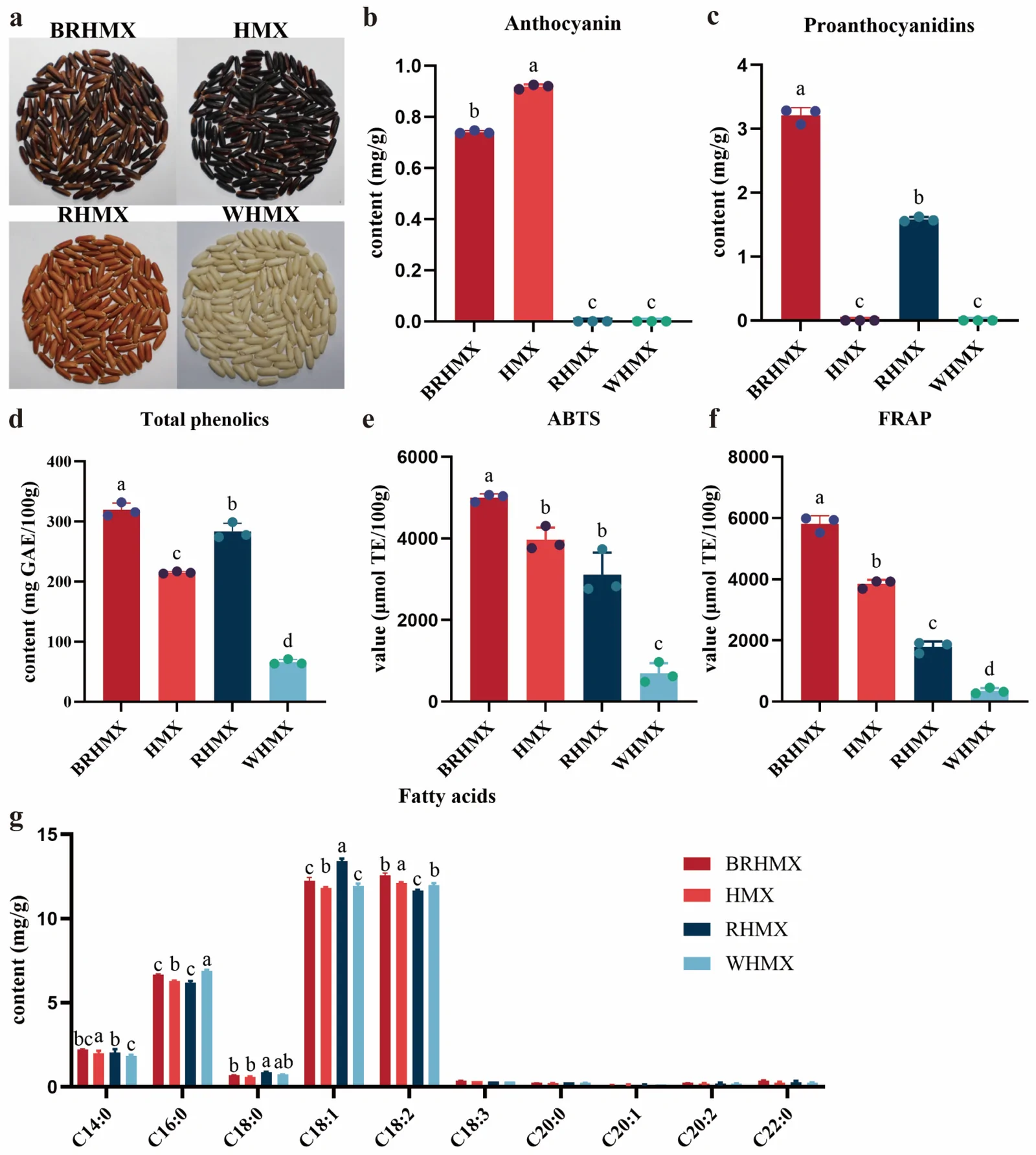
Grain appearance, major nutritional and bioactive components, and chemical antioxidant capacity of the four Huamoxiang lines with different pericarp colors. (**a**) Grain appearance. (**b**) Anthocyanin content. (**c**) Proanthocyanidin content. (**d**) Total phenolic content. (**e**) ABTS antioxidant capacity. (**f**) FRAP antioxidant capacity. (**g**) Fatty acid composition. GAE, gallic acid equivalents. TE, Trolox equivalents. Data are presented as means ± SD (*n* = 3). Different lowercase letters indicate significant differences among the four lines (*p* < 0.05), as determined by Tukey’s HSD test.

Finally, four Huamoxiang lines with highly similar genetic backgrounds but distinct pericarp colors were developed (Figure 1a). Genome-wide SNP chip analysis showed that BRHMX exhibited the highest genetic similarity to HMX (98.04%), followed by WHMX (95.76%) and RHMX (93.56%) (Figure S1).

### 3.2. Analysis of Grain Size, Pigment Content, Major Nutritional Components, Total Phenolics, and Total Antioxidant Capacity

Analysis of pigment contents revealed distinct differences among the four lines (Figure 1b,c). HMX accumulated 0.92 mg/g anthocyanins but lacked detectable proanthocyanidins, whereas BRHMX accumulated both anthocyanins (0.74 mg/g) and proanthocyanidins (3.21 mg/g). RHMX contained 1.58 mg/g proanthocyanidins but no anthocyanins, while WHMX lacked both anthocyanins and proanthocyanidins. Compared with HMX, BRHMX retained 80.4% of the anthocyanin content and showed a 103% increase in proanthocyanidin content relative to RHMX, indicating the simultaneous accumulation of both pigment types in BRHMX.

To evaluate whether pericarp pigmentation affected basic grain quality traits, we compared grain morphology and major nutritional components among the four lines. WHMX showed a slightly higher average grain length and width, yet no significant differences in grain size were detected across the four lines. Similarly, total starch, amylose content, total protein, and the composition of 17 amino acids showed no significant differences (Tables S2 and S3), indicating that differences in pericarp pigmentation were associated with limited variation in these major grain nutritional components.

In contrast, differences were observed in fatty acid composition. BRHMX and RHMX showed increased proportions of unsaturated fatty acids, particularly oleic acid (C18:1) and linoleic acid (C18:2), compared with HMX, whereas WHMX exhibited a higher proportion of palmitic acid (C16:0) (Figure 1g). These results indicate differences in specific fatty acid profiles among the four lines, although the underlying regulatory mechanisms require further investigation.

Evaluation of phenolic compounds further highlighted the nutritional advantages of the dual-pigmented line. BRHMX exhibited the highest total phenolic content among the four lines, followed by RHMX and HMX, whereas WHMX showed the lowest level (Figure 1d). Among the 50 soluble phenolic acids quantified, BRHMX showed the highest accumulation of 31 compounds, while HMX and RHMX showed the highest levels of 8 and 9 compounds, respectively. In contrast, WHMX exhibited the highest abundance of only two phenolic acids and showed the lowest levels for 35 compounds (Table S4).

Consistent with the differences in phenolic composition, BRHMX displayed the highest chemical antioxidant capacity compared with the other lines. Its ABTS and FRAP values were 1.25-fold (*p* = 0.0236) and 1.51-fold (*p* < 0.0001) higher than those of HMX, respectively. RHMX exhibited reduced antioxidant capacity compared with HMX, with decreases of 21.68% in ABTS activity (*p* = 0.0551) and 53.54% in FRAP activity (*p* < 0.0001). WHMX showed the lowest antioxidant capacity, retaining only 17.48% of the ABTS activity and 9.12% of the FRAP activity of HMX (*p* < 0.0001) (Figure 1e,f). Collectively, these results indicate that BRHMX combined the accumulation of both anthocyanins and proanthocyanidins with higher phenolic content and chemical antioxidant capacity.

To further evaluate the effects of cooking, the contents of anthocyanins, proanthocyanidins, and total phenolics, together with chemical antioxidant capacity, were determined in cooked rice (Figure S2). Cooking significantly reduced the anthocyanin contents of BRHMX and HMX by 48.65% and 51.09%, respectively, while the proanthocyanidin contents of BRHMX and RHMX decreased by 67.29% and 67.72%, respectively. The total phenolic content decreased by 60.93%, 24.07%, 58.43%, and 39.43% in BRHMX, HMX, RHMX, and WHMX, respectively. Similarly, ABTS antioxidant capacity decreased by 48.85%, 29.59%, 60.94%, and 89.17% in BRHMX, HMX, RHMX, and WHMX, respectively. FRAP antioxidant capacity decreased by 47.20%, 10.20%, and 55.10% in BRHMX, HMX, and RHMX, respectively, whereas the 7.97% decrease in WHMX was not significant. Overall, cooking resulted in variable reductions in bioactive compounds and chemical antioxidant capacity among the four Huamoxiang lines.

### 3.3. Metabolome Analysis of Mature Seeds

**Figure 2:**
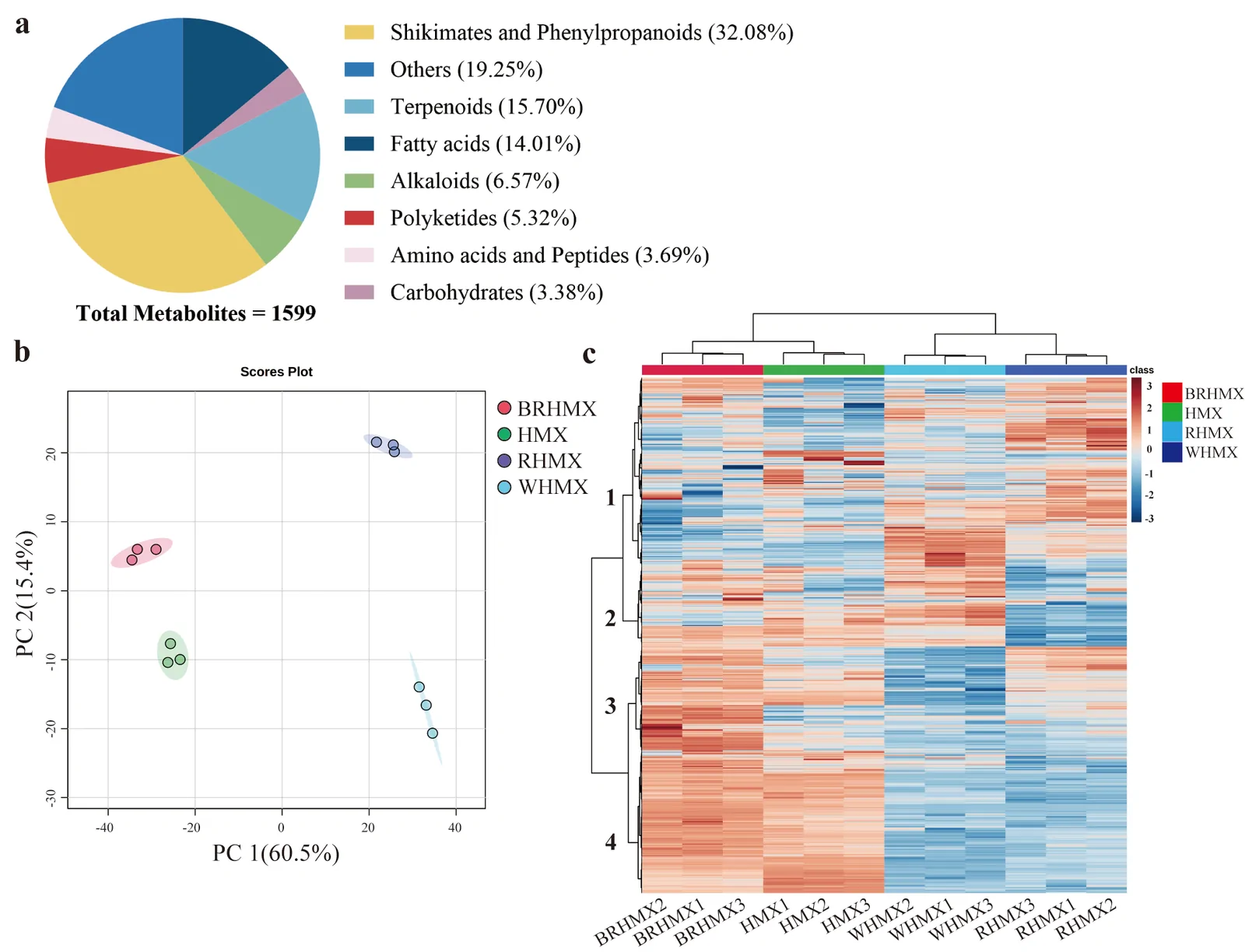
Metabolomic profiles of mature seeds from the four Huamoxiang lines. (**a**) The classification of annotated metabolites. (**b**) The PCA score plot based on metabolomic profiles. (**c**) HCA of metabolites among the four lines.

Secondary metabolites, particularly flavonoids and phenolic compounds, are important contributors to the distinctive composition of pigmented rice [22,51,52]. To characterize metabolic differences associated with pericarp pigmentation, untargeted metabolomic profiling was performed on mature seeds of the four lines. A total of 1599 Level 1 and Level 2 metabolites were classified into eight major chemical super classes (Figure 2a). Among these, shikimate and phenylpropanoid compounds represented the largest proportion (32.08%), followed by terpenoids (15.70%) and fatty acids (14.01%). Within the shikimate and phenylpropanoid categories, 51 phenolic acids and 263 flavonoids were detected, including 9 anthocyanins and 10 proanthocyanidins, with distinct accumulation patterns among the four lines (Table S5).

Multivariate statistical analysis showed distinct metabolic profiles among the four Huamoxiang lines. PCA showed that PC1 and PC2 explained 60.5% and 15.4% of the total variance, respectively, and revealed clear differences in metabolic profiles among the four lines (Figure 2b). HCA further classified the metabolites into four major groups, consistent with the differences in metabolite accumulation among the lines (Figure 2c).

**Figure 3:**
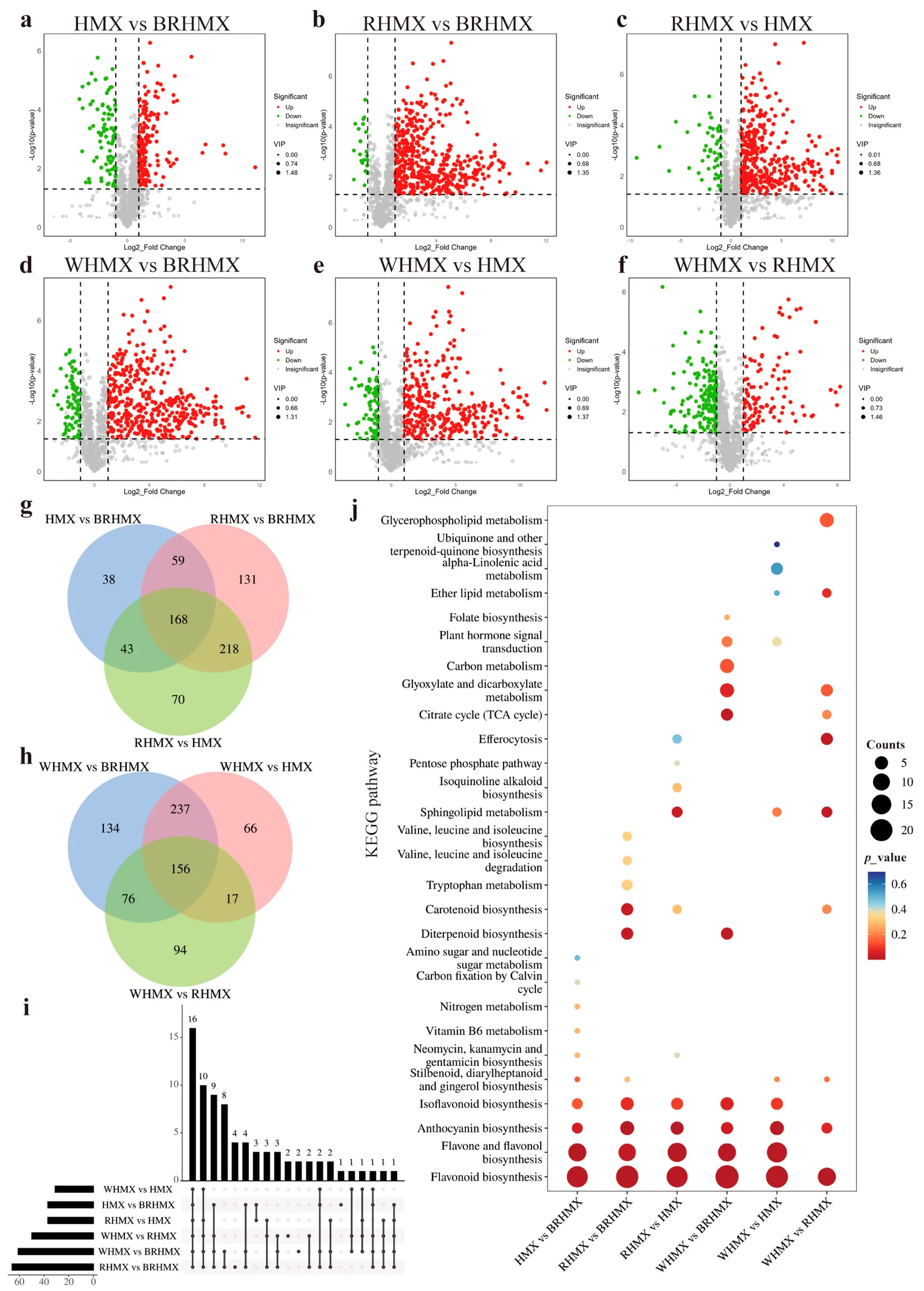
Differential metabolite accumulation analysis of mature seeds from the four Huamoxiang lines. Volcano plots showing DAMs in the following pairwise comparisons: (**a**) HMX vs. BRHMX, (**b**) RHMX vs. BRHMX, (**c**) RHMX vs. HMX, (**d**) WHMX vs. BRHMX, (**e**) WHMX vs. HMX, and (**f**) WHMX vs. RHMX. Red and green dots indicate significantly upregulated and downregulated metabolites, respectively. Panels (**g**,**h**) show shared and unique DAMs among the pigmented Huamoxiang lines and between the pigmented lines and WHMX, respectively. (**i**) The UpSet plot shows KEGG pathway enrichment patterns of DAMs. Bars represent the number of enriched pathways in each comparison or intersection, and connected dots indicate the corresponding comparison groups. (**j**) KEGG pathway enrichment analysis of DAMs. Bubble size represents the number of DAMs enriched in each pathway, and color intensity indicates the significance of enrichment.

OPLS-DA was subsequently performed for six pairwise comparisons. All models showed R^2^Y and Q^2^ values above 0.9 under seven-fold cross-validation, and 200-permutation tests further supported the robustness of the models (*p* < 0.05) (Figure S3). Differentially accumulated metabolites (DAMs) were identified using the criteria of VIP ≥ 1, fold change (FC) ≥ 2 or FC ≤ 0.5, and *p* < 0.05. The number of DAMs varied among different comparisons, with 203 upregulated and 105 downregulated metabolites in HMX versus BRHMX, 554 upregulated and 22 downregulated metabolites in RHMX versus BRHMX, and 443 upregulated and 56 downregulated metabolites in RHMX versus HMX. Comparisons involving WHMX also showed substantial metabolic differences (Figure 3a–f). Notably, BRHMX showed a greater number of metabolites with higher accumulation relative to the other lines, indicating substantial metabolic differences associated with the dual-pigmented line.

Flavonoid metabolites accounted for 25.95–35.71% of the DAMs across all comparisons, and more than half of the shared DAMs belonged to metabolites associated with shikimate and phenylpropanoid metabolism (Figure 3g,h). These results indicate that flavonoid-related metabolites contributed substantially to the metabolic differences among the four Huamoxiang lines.

KEGG pathway enrichment analysis showed that DAMs were mainly associated with secondary metabolic pathways. Comparisons among the pigmented lines resulted in enrichment of 37, 66, and 37 pathways, whereas comparisons between pigmented lines and WHMX resulted in enrichment of 61, 31, and 50 pathways, respectively (Figure 3i; Table S6). Flavonoid biosynthesis, flavonol biosynthesis, and anthocyanin biosynthesis were consistently enriched across all comparison groups (*p* < 0.05) (Figure 3j), suggesting that flavonoid metabolism was closely associated with the observed metabolic differences related to pericarp pigmentation. The distinct metabolic profile of BRHMX provides a basis for further investigation of the metabolic consequences of the simultaneous accumulation of anthocyanins and proanthocyanidins.

### 3.4. Integrated Metabolomic and Transcriptomic Profiling of 10 DAP Pericarps

**Figure 4:**
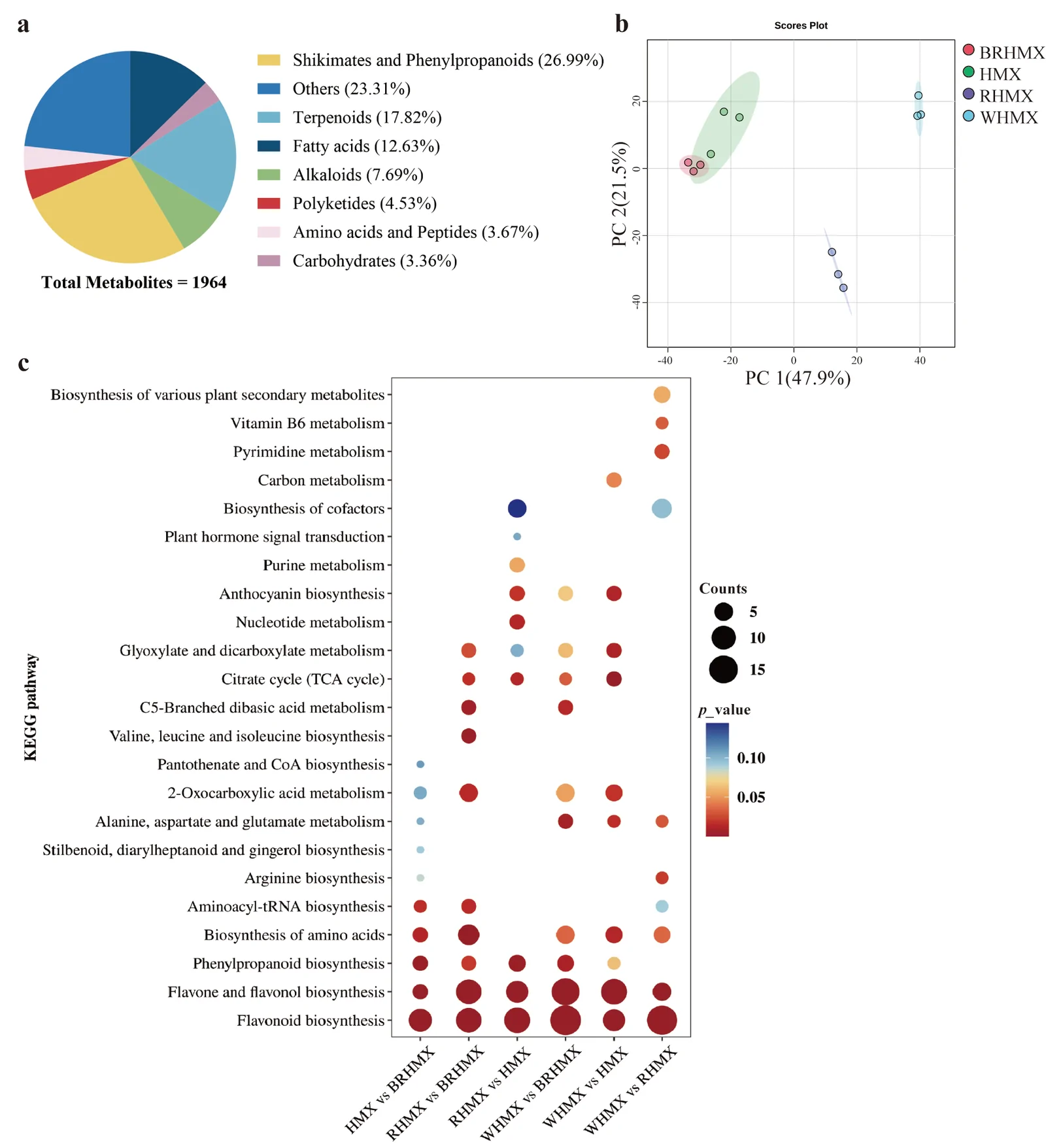
Metabolomic profiling and differential metabolite analysis of 10 DAP pericarps from the four Huamoxiang lines. (**a**) Classification of annotated metabolites identified in the 10 DAP pericarps. (**b**) PCA score plot based on metabolomic profiles. (**c**) KEGG pathway enrichment analysis of DAMs. Bubble size represents the number of DAMs enriched in each pathway, and color intensity indicates the significance of pathway enrichment.

The pericarp at 10 DAP represents an important developmental stage associated with active flavonoid biosynthesis and pigment accumulation [53,54]. To investigate the early metabolic changes associated with pericarp color formation, pericarps from the four Huamoxiang lines were collected at this stage for untargeted metabolomic analysis (Figure 4a). PCA showed that PC1 and PC2 explained 47.9% and 21.5% of the total variance, respectively. RHMX and WHMX were clearly separated from each other and from HMX and BRHMX, whereas BRHMX and HMX showed a close distribution pattern and were difficult to distinguish based on their metabolite profiles (Figure 4b). This result suggested that BRHMX and HMX retained similar metabolic characteristics during early pericarp development, which was further supported by pairwise differential metabolite analysis (Figure S4). KEGG enrichment analysis showed that the DAMs identified among the four lines were mainly associated with flavonoid biosynthesis and phenylpropanoid biosynthesis pathways (Figure 4c).

Transcriptome analysis of pericarps at the same developmental stage further revealed transcriptional differences associated with pigment formation. Among the pairwise comparisons, HMX versus BRHMX showed the fewest differentially expressed genes (DEGs), consistent with their similar metabolomic profiles (Figure S5). KEGG enrichment analysis revealed that DEGs among the four lines were significantly enriched in flavonoid biosynthesis-related pathways to different extents (Figure S6). Several structural genes involved in flavonoid biosynthesis, including *CHI6*, *CHS*, and *F3’H*, showed relatively high expression levels in both HMX and BRHMX (Figure S7). Although HMX carries a non-functional *Rc* allele, *rc* transcripts were still detected at a level comparable to that in BRHMX. Similarly, *LAR* expression was detectable in HMX. However, the presence of the functional *Rc* allele in BRHMX was associated with increased *LAR* expression (Figure S7), which corresponded with the enhanced proanthocyanidin accumulation observed in BRHMX.

**Figure 5:**
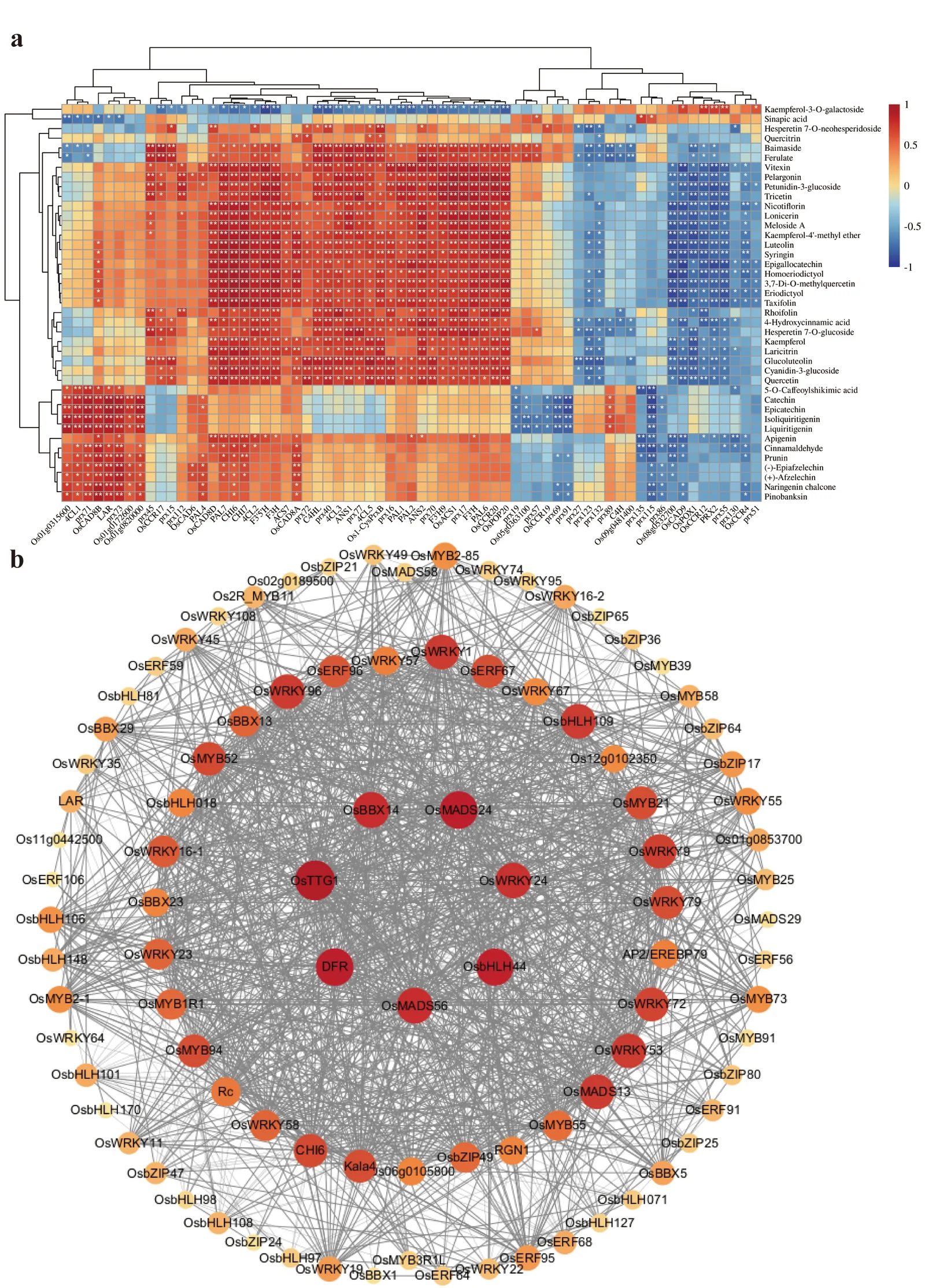
Integrated transcriptomic and metabolomic analysis of 10 DAP pericarps from the four Huamoxiang lines. (**a**) Correlation clustering heatmap of flavonoid-related metabolites and differentially expressed genes co-enriched in flavonoid and anthocyanin biosynthetic pathways. Color intensity represents the correlation coefficient, with red and blue indicating positive and negative correlations, respectively. *, *p* < 0.05; **, *p* < 0.01. (**b**) Correlation network of differentially expressed transcription factors and key genes involved in anthocyanin and proanthocyanidin biosynthesis. Edges represent significant correlations, and edge thickness indicates the strength of the association. Node size represents connectivity, and node color indicates different gene categories.

To examine the association between gene expression and metabolite accumulation, Pearson’s correlation analysis was performed using 67 DEGs and 41 DAMs involved in flavonoid and anthocyanin biosynthesis pathways (*p* < 0.05) (Figure 5a). Cyanidin-3-*O*-glucoside showed positive correlations with several structural genes, including *PAL*, *CHI6*, and *ANS*, as well as peroxidase genes such as *Prx77* and *Prx17*. In contrast, catechin and epicatechin were positively correlated with *LAR* and peroxidase genes including *Prx73* and *Prx71*, but negatively correlated with *Prx115*, *Prx69*, and *Prx51*. These correlation patterns indicated distinct associations between gene expression and the accumulation of anthocyanins and proanthocyanidins during early pericarp development.

Furthermore, transcription factors from the MYB, BBX, WRKY, and MADS families were analyzed to identify potential regulators involved in flavonoid biosynthesis. A correlation network was constructed based on six key genes (*Rc*, *Kala4*, *OsTTG1*, *CHI*, *DFR*, and *LAR*) using the criteria of |r| ≥ 0.8 and *p* < 0.05 (Figure 5b and Figure S8). *OsTTG1* showed the highest connectivity within the network, indicating that its expression was strongly associated with multiple flavonoid-related genes. In addition, *OsBBX14* and *OsMADS24* showed strong associations with *Kala4*, *OsTTG1*, and *DFR*, suggesting that they may be candidate transcription factors associated with flavonoid biosynthesis.

## 4. Discussion

The increasing demand for healthier dietary choices has encouraged the development of whole-grain foods with added nutritional value. The “National Whole Grain Action Plan (2024–2035)” proposed by the Chinese government highlights the importance of increasing whole-grain consumption among residents. Black rice and red rice are important pigmented whole-grain resources because they retain dietary fiber, proteins, lipids, vitamins, minerals, and bioactive pigments that are largely removed during milling and polishing [55,56]. Anthocyanins in black rice and proanthocyanidins in red rice are recognized as important phenolic compounds associated with antioxidant and other health-related properties [27,28]. However, these two pigment types are usually present separately in rice germplasm. Therefore, dual-pigmented rice, which simultaneously accumulates anthocyanins and proanthocyanidins, provides an opportunity to combine the nutritional characteristics of both pigmented rice types.

Although the enrichment of flavonoid-, flavonol-, and anthocyanin-related pathways among the DAMs was expected given the distinct pigmentation of the four Huamoxiang lines, the comparative value of the present system lies in examining effects that extend beyond simply confirming the known biochemical basis of pigmentation. In particular, BRHMX provides an opportunity to examine the metabolic consequences of combining anthocyanin and proanthocyanidin accumulation within a highly similar genetic background. The accumulation of anthocyanins and proanthocyanidins in rice pericarps is mainly determined by key regulatory genes, including *Rc* and *Kala4* [17,18,57]. Functional alleles of these genes occur at relatively low frequencies in rice populations, which may account for the limited occurrence of dual-pigmented rice. Because anthocyanin and proanthocyanidin biosynthesis share common upstream metabolic pathways, activation of both branches could alter the distribution of shared metabolic intermediates. Notably, the metabolic outcome observed in BRHMX was not simply intermediate between those of conventional black and red rice. BRHMX maintained a high anthocyanin level while showing a moderate reduction compared with HMX, accompanied by a more than two-fold increase in proanthocyanidin content compared with RHMX. These results suggest that the introduction of the functional *Rc* allele into the HMX background may contribute to enhanced proanthocyanidin accumulation without eliminating anthocyanin accumulation. This pattern suggests a potential redistribution of shared flavonoid precursors toward proanthocyanidin biosynthesis while substantial anthocyanin accumulation is maintained, although further validation is required.

Transcriptome analysis of 10 DAP pericarps provided further evidence for this interpretation. HMX and BRHMX exhibited similar expression patterns for several upstream flavonoid biosynthetic genes, including *CHI6*, *CHS*, and *F3’H*, indicating that the activation of the upstream flavonoid pathway characteristic of the black rice background was largely maintained in BRHMX. Meanwhile, the functional *Rc* allele in BRHMX was associated with increased *LAR* expression, which was consistent with enhanced proanthocyanidin accumulation. The combined metabolomic and transcriptomic patterns are therefore consistent with a model in which functional *Kala4* and *Rc* support the production of both anthocyanins and proanthocyanidins from shared flavonoid precursors. Consequently, BRHMX exhibited the highest total phenolic content and chemical antioxidant capacity among the four lines before cooking. These results suggest that the metabolic differences associated with pericarp pigmentation extend beyond the presence or absence of individual pigment classes. The combination of anthocyanin and proanthocyanidin accumulation in BRHMX was associated with enhanced phenolic accumulation and chemical antioxidant capacity. Considering its close genetic similarity to the elite black rice cultivar HMX, BRHMX may therefore serve as a useful material for studying and improving the nutritional and functional properties of pigmented whole-grain rice.

The integrated metabolomic and transcriptomic analyses also identified several genes whose expression patterns were closely associated with flavonoid-related metabolic variation. In addition to the established regulatory genes *Rc* and *Kala4*, several transcription factors, including *OsTTG1*, *OsBBX14*, and *OsMADS24*, showed strong correlations with flavonoid biosynthetic genes and may represent candidate regulators potentially involved in pigment accumulation [53,54]. The correlation analysis also revealed heterogeneous relationships among peroxidase genes and pigment-related metabolites. For example, *Prx77* was positively associated with anthocyanin-related metabolites, whereas *Prx123* showed an opposite pattern. Peroxidases have been implicated in flavonoid metabolism and, in some cases, anthocyanin degradation [58,59], while different members of the class III peroxidase family may show distinct relationships with pigmentation [60]. These findings provide several candidates for further investigation of flavonoid metabolism in rice pericarps. However, the observed correlations do not establish direct regulatory or functional relationships, and further functional studies will be required to determine whether these genes contribute to pigment accumulation.

Although statistically significant differences were observed in the profiles of several fatty acids among the four Huamoxiang lines, these differences should be interpreted cautiously. Because rice grains contain relatively low amounts of total lipids, variation in individual fatty acids is unlikely to result in substantial differences in the overall nutritional composition or dietary fatty acid intake of the four lines. Compared with these relatively limited differences in fatty acid composition, the more pronounced variation in phenolic compounds and chemical antioxidant capacity was closely associated with pericarp pigmentation. These differences, however, may be altered during cooking, which is an important consideration for rice intended for consumption.

Cooking substantially affected the retention of bioactive compounds and chemical antioxidant capacity in the four Huamoxiang lines. In the present study, cooking markedly reduced anthocyanins, proanthocyanidins, total phenolics, and chemical antioxidant capacity, although the extent of these changes varied among lines and compounds. Similar reductions in anthocyanins and phenolic compounds have been reported in cooked black rice, with the magnitude of change depending on cooking conditions and rice genotype. Anthocyanin losses of 65.4–79.8% have been reported under different cooking methods, accompanied by a 2.7–3.4-fold increase in protocatechuic acid, suggesting that thermal degradation of anthocyanins may be associated with changes in other phenolic compounds [61]. Previous studies have also reported substantial reductions in proanthocyanidins after cooking, with an average decrease of approximately 39% under different cooking methods [62]. In the present study, proanthocyanidins decreased by 67.29% and 67.72% in BRHMX and RHMX, respectively, and the variation in degradation rates may partly reflect differences in cooking equipment, cooking time, and temperature. Other studies have also shown that cooking can substantially alter anthocyanin and phenolic profiles and chemical antioxidant capacity in black rice, with the extent of these changes depending on the cooking method and rice genotype [62,63,64,65].

Importantly, ABTS and FRAP reflect chemical antioxidant capacity under in vitro conditions and do not directly demonstrate biological antioxidant activity or health effects. Because no in vivo antioxidant assays were performed in the present study, the observed differences cannot be directly extrapolated to physiological effects. Although BRHMX showed attenuated chemical antioxidant capacity after cooking, it retained both anthocyanins and proanthocyanidins, distinguishing it from the single-pigment lines. The degradation rates of anthocyanins and proanthocyanidins in BRHMX were comparable to those in the corresponding single-pigment lines, suggesting that the simultaneous presence of both pigment classes did not confer a detectable protective effect against thermal degradation under the conditions tested in this study. However, these observations do not exclude the possibility of a copigmentation effect, as the present experimental design was not specifically optimized to detect such interactions. Further studies using purified compounds or controlled mixtures, together with spectroscopic and stability analyses, would be required to directly evaluate whether copigmentation contributes to the thermal stability of the dual-pigmented system. The effects of cooking-induced changes in phenolic composition on digestion, bioaccessibility, bioavailability, and bioactivity also warrant further investigation.

Finally, two limitations of the present study should be addressed in the near future. First, the essential amino acid tryptophan was not quantified because it is degraded under the acid hydrolysis conditions used for amino acid analysis; future work should therefore employ alkaline or enzymatic hydrolysis followed by HPLC determination. Second, antioxidant activity was assessed only by chemical assays under in vitro conditions; future studies should evaluate antioxidant activity under physiologically relevant conditions, such as cell-based or in vivo models, to better assess the biological relevance of the observed chemical antioxidant capacity.

## 5. Conclusions

This study characterized four genetically similar Huamoxiang rice lines with distinct pericarp pigmentation and systematically evaluated their nutritional composition, bioactive compounds, chemical antioxidant capacity, and associated metabolic regulation. The results showed that differences in pericarp pigmentation had limited effects on grain morphology and major nutritional components, including starch, protein, and amino acids. In contrast, the dual-pigmented line BRHMX, carrying functional Rc and Kala4 alleles, exhibited enhanced accumulation of proanthocyanidins and total phenolics and higher chemical antioxidant capacity before cooking, while retaining a substantial level of anthocyanins. Integrated metabolomic and transcriptomic analyses further indicated that flavonoid-related metabolic pathways were closely associated with pericarp color variation and coordinated changes in structural genes, transcription factors, and other regulatory components. Cooking substantially reduced anthocyanins, proanthocyanidins, total phenolics, and chemical antioxidant capacity, although the magnitude of these changes varied among the four lines. Overall, the results provide a comprehensive understanding of the metabolic basis and nutritional characteristics associated with pericarp pigmentation in Huamoxiang rice and highlight BRHMX as a promising genetic resource for the development and utilization of dual-pigmented whole-grain rice.

## Data Availability

The original contributions presented in the study are included in the article and Supplementary Materials, further inquiries can be directed to the corresponding author.

## Supplementary Materials

The following supporting information can be downloaded at: https://www.mdpi.com/article/10.3390/foods15193530/s1, Figure S1. Development and genetic background characterization of the Huamoxiang lines with different pericarp colors; Figure S2. Bioactive components and chemical antioxidant capacity of cooked rice from the four Huamoxiang lines with different pericarp colors; Figure S3. OPLS-DA score plots and permutation tests for pairwise metabolomic comparisons of mature seeds among the four Huamoxiang lines; Figure S4. DAM analysis of 10 DAP pericarp metabolomes among the four Huamoxiang lines; Figure S5. DEG analysis of 10 DAP pericarp transcriptomes among the four Huamoxiang lines; Figure S6. KEGG pathway enrichment analysis of DEGs identified from 10 DAP pericarp transcriptomes of the four Huamoxiang lines; Figure S7. Expression profiles of genes involved in anthocyanin and proanthocyanidin biosynthesis and regulation in 10 DAP pericarps of the four Huamoxiang lines; Figure S8. Correlation heatmap showing associations between transcription factors and key genes involved in anthocyanin and proanthocyanidin biosynthesis in 10 DAP pericarps of the four Huamoxiang lines; Table S1. The primer sequences used in this study; Table S2. Appearance, grain size, major nutritional component contents, and genetic similarity of mature seeds from the four Huamoxiang lines; Table S3. 17 kinds of amino acids content (%) of mature seeds from the four Huamoxiang lines; Table S4. Abundance of soluble phenolic acids of mature seeds from the four Huamoxiang lines; Table S5: Metabolomic profiles of flavonoids and phenolic acids in mature seeds of four Huamoxiang rice lines (Note: Sheet 1: All detected metabolites; Sheet 2: Flavonoid and phenolic acids); Table S6: KEGG enrichment analysis of DAMs derived from six pairwise comparisons of mature seeds of four Huamoxiang lines (Note: Sheet 1: HMX vs. BRHMX; Sheet 2: HMX vs. RHMX; Sheet 3: RHMX vs. BRHMX; Sheet 4: WHMX vs. BRHMX; Sheet 5: WHMX vs. HMX; Sheet 6: WHMX vs. RHMX).
