# Supplementary material for: Dual-Pigmented Rice: A Dual-Pigmented Whole Grain with Enhanced Nutritional and Antioxidant Properties
Source: Foods. 2026 Oct 2;15(19):3530. doi: 10.3390/foods15193530 (PMC13664859; doi:10.3390/foods15193530)
Supplement: Supplementary file 1 [file foods-15-03530-s001.zip › foods-4525104-supplementary.pdf]

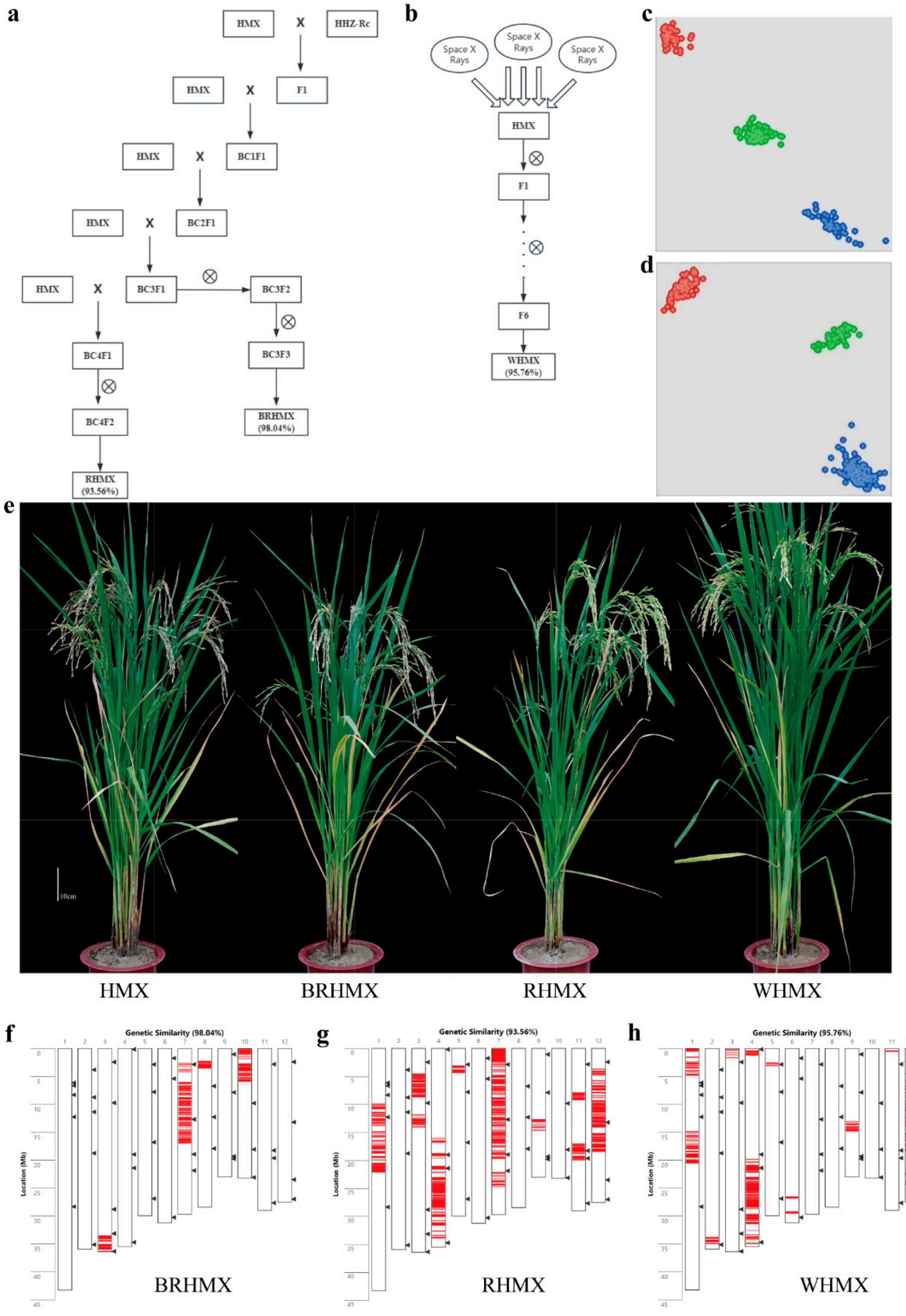

Figure S1. Development and genetic background characterization of the Huamoxiang lines with different pericarp colors. (a) Breeding schemes for the development of BRHMX and RHMX. (b) Breeding process of WHMX. HHZ-Rc represents the Huanghuazhan *Rc* single gene introgression line. "×" indicates hybridization, and "⊗"

indicates self-pollination. (c,d) KASP genotyping of *Kala4* (c) and *Rc* (d). Red, blue, and green dots represent homozygous functional alleles, homozygous non-functional alleles, and heterozygous genotypes, respectively. (e) Plant architecture of the four Huamoxiang lines. Scale bar = 10 cm. (f–h) Genome-wide genetic similarity analysis of BRHMX (f), RHMX (g), and WHMX (h) compared with HMX. Red lines indicate genomic regions with genotype differences relative to HMX, and triangles indicate the positions of 48 national standard rice SSR markers.

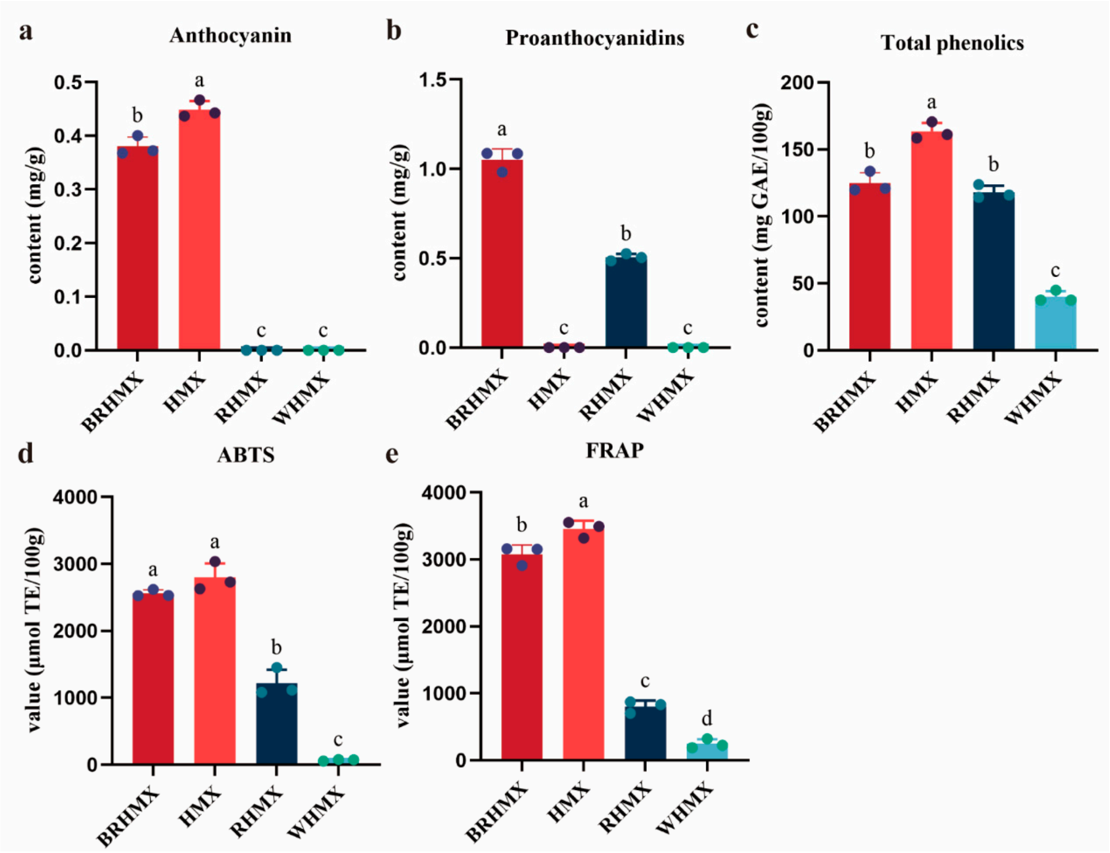

Figure S2. Bioactive components and chemical antioxidant capacity of cooked rice from the four Huamoxiang lines with different pericarp colors. (a) Anthocyanin content, (b) proanthocyanidin content, (c) total phenolic content, (d) ABTS antioxidant capacity, and (e) FRAP antioxidant capacity. Data are presented as mean ± SD (n = 3), and different lowercase letters indicate significant differences among lines according to Tukey's HSD test ($P < 0.05$).

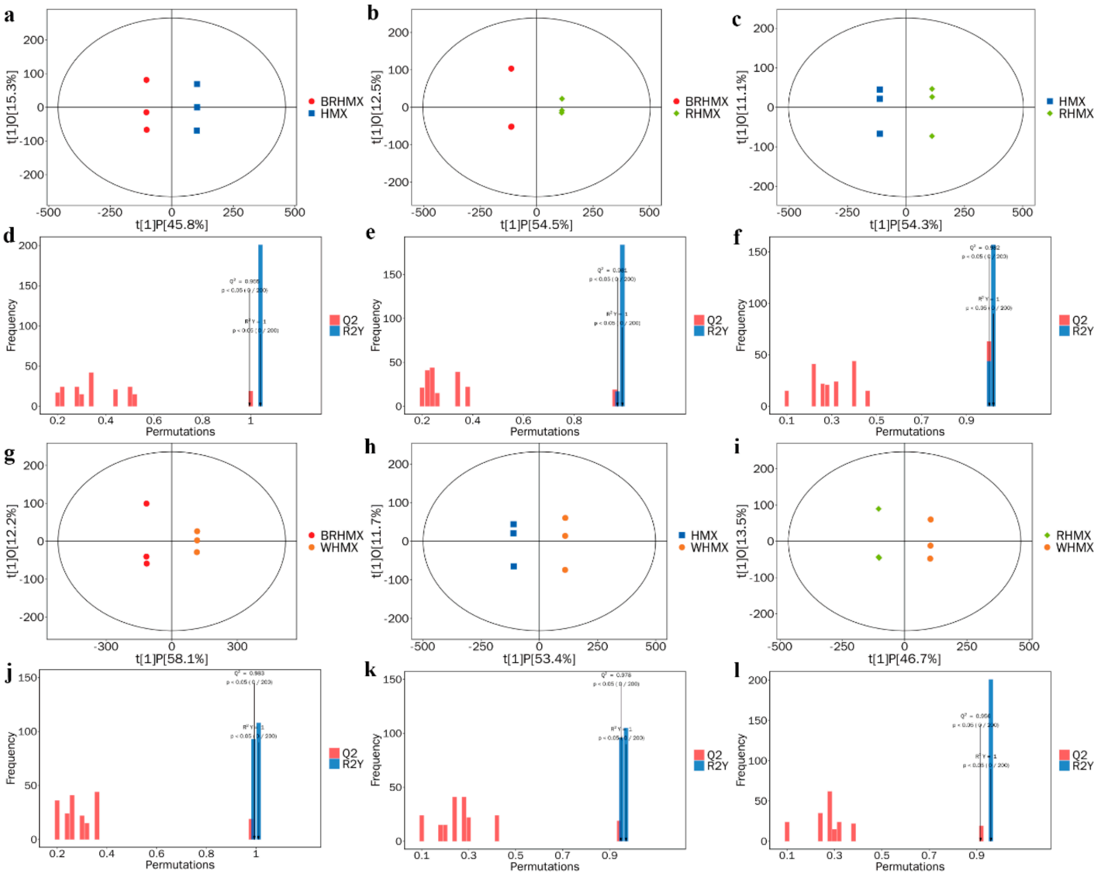

Figure S3. OPLS-DA score plots and permutation tests for pairwise metabolomic comparisons of mature seeds among the four Huamoxiang lines. (a–c, g–i) OPLS-DA score plots of HMX vs. BRHMX, RHMX vs. BRHMX, RHMX vs. HMX, WHMX vs. BRHMX, WHMX vs. HMX, and WHMX vs. RHMX, respectively. (d–f, j–l) Corresponding 200-permutation validation plots for each pairwise comparison. R^2Y represents the proportion of variance explained by the model, and Q^2 indicates the predictive ability of the model.

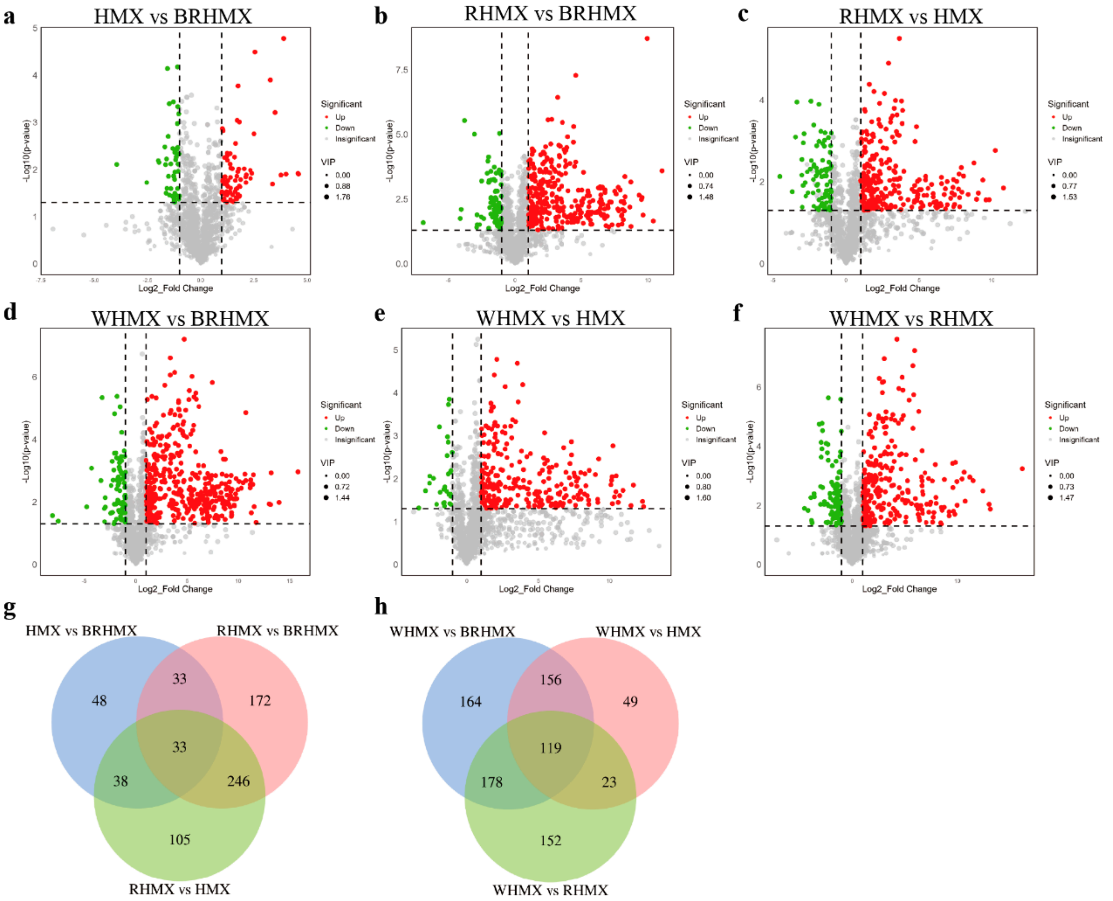

Figure S4. DAM analysis of 10 DAP pericarp metabolomes among the four Huamoxiang lines. (a–f) Volcano plots showing DAMs identified in pairwise comparisons of HMX vs. BRHMX, RHMX vs. BRHMX, RHMX vs. HMX, WHMX vs. BRHMX, WHMX vs. HMX, and WHMX vs. RHMX, respectively. Red, green, and gray dots indicate significantly upregulated metabolites, significantly downregulated metabolites, and metabolites without significant differences, respectively. (g) Venn diagram showing shared and unique DAMs among the pigmented Huamoxiang lines. (h) Venn diagram showing shared and unique DAMs between the pigmented Huamoxiang lines and WHMX.

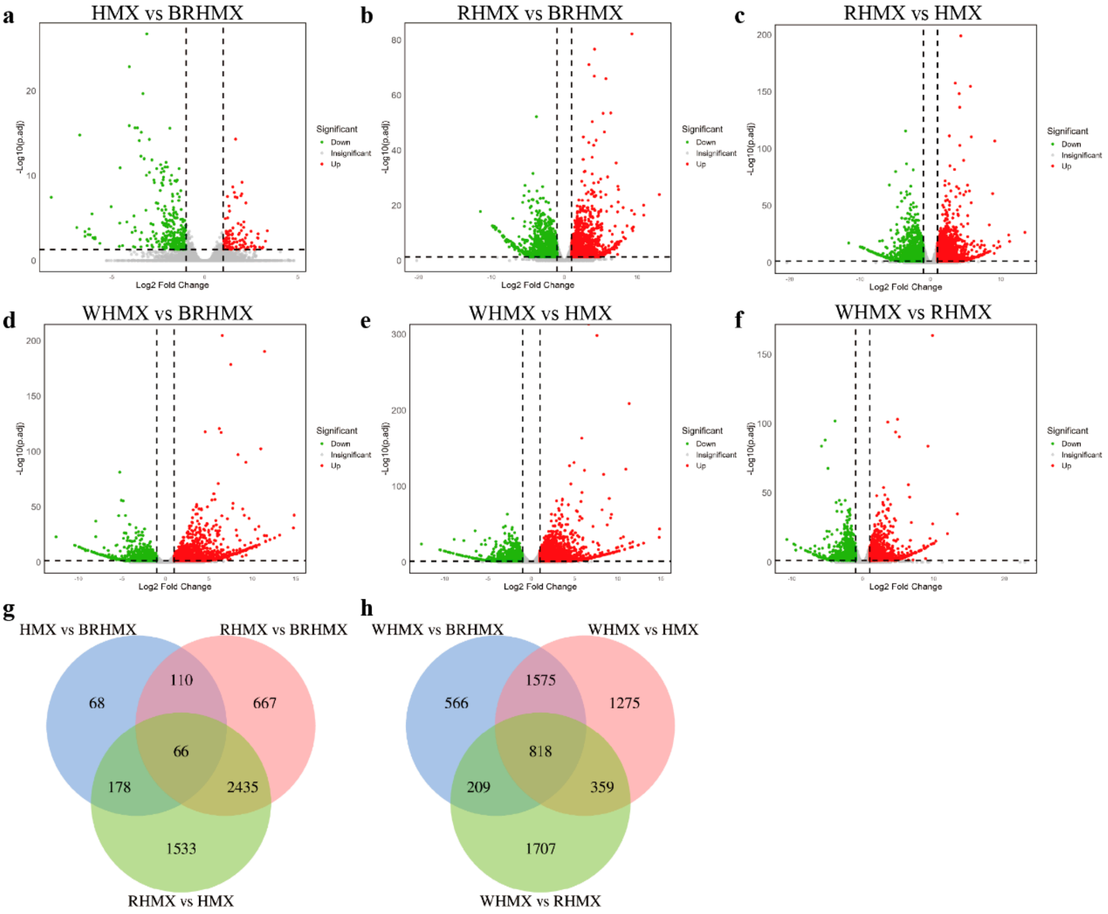

Figure S5. DEG analysis of 10 DAP pericarp transcriptomes among the four Huamoxiang lines. (a–f) Volcano plots showing DEGs identified in pairwise comparisons of HMX vs. BRHMX, RHMX vs. BRHMX, RHMX vs. HMX, WHMX vs. BRHMX, WHMX vs. HMX, and WHMX vs. RHMX, respectively. Red, green, and gray dots indicate significantly upregulated genes, significantly downregulated genes, and genes without significant differential expression, respectively. (g) Venn diagram showing shared and unique DEGs among the pigmented Huamoxiang lines. (h) Venn diagram showing shared and unique DEGs between the pigmented Huamoxiang lines and WHMX.

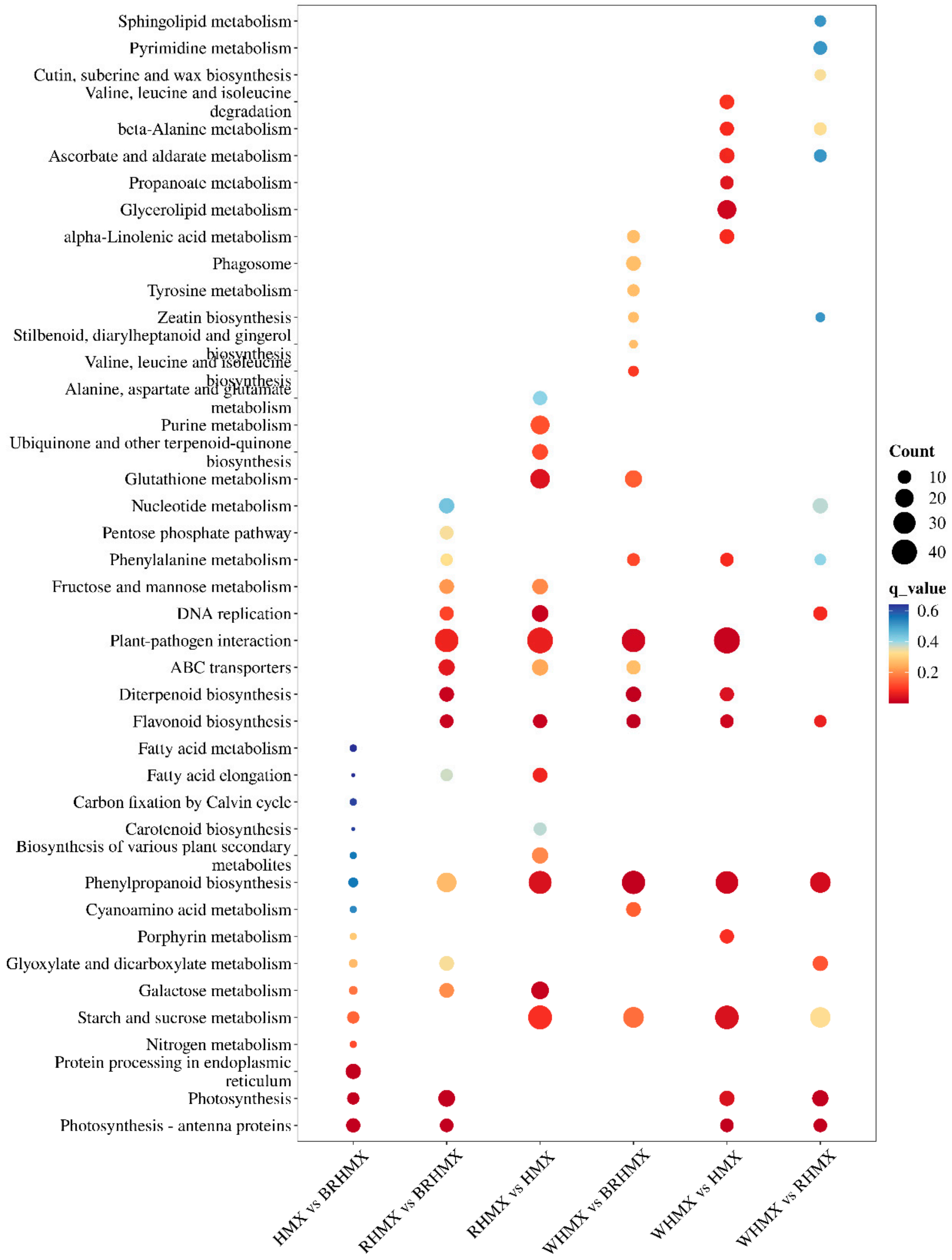

Figure S6. KEGG pathway enrichment analysis of DEGs identified from 10 DAP pericarp transcriptomes of the four Huamoxiang lines. Bubble size represents the number of DEGs enriched in each pathway, and color intensity indicates the significance level of pathway enrichment.

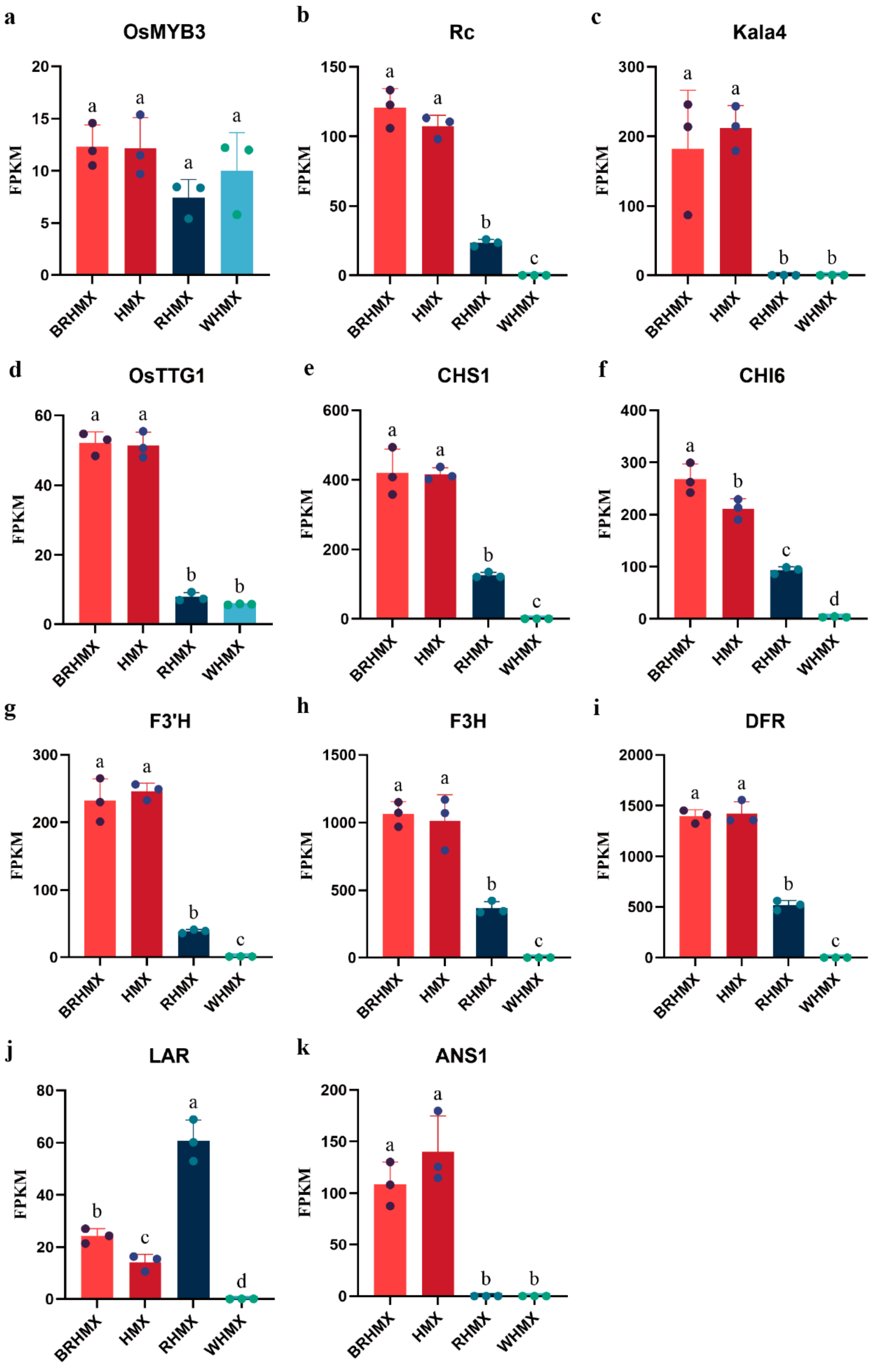

Figure S7. Expression profiles of genes involved in anthocyanin and proanthocyanidin biosynthesis and regulation in 10 DAP pericarps of the four Huamoxiang lines. Gene

expression levels are shown as FPKM values. Data are presented as mean ± SD (n = 3), and different lowercase letters indicate significant differences among lines according to Tukey's HSD test ($P < 0.05$).

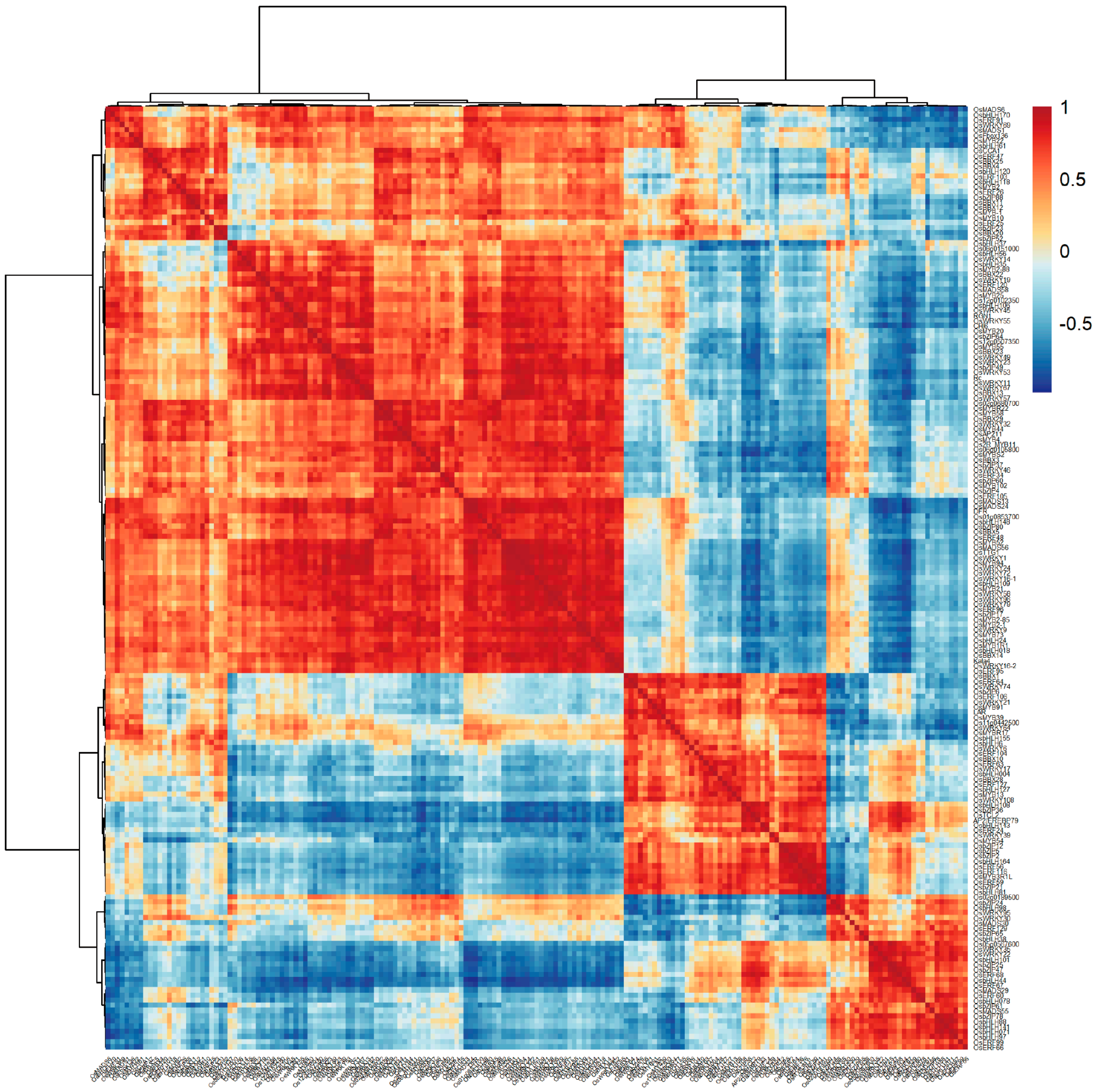

Figure S8. Correlation heatmap showing associations between transcription factors and key genes involved in anthocyanin and proanthocyanidin biosynthesis in 10 DAP pericarps of the four Huamoxiang lines. Correlation coefficients were calculated using Pearson's correlation analysis, and hierarchical clustering was performed using the Ward.D method. Color intensity represents the strength of the correlation, with red indicating positive correlations and blue indicating negative correlations.
